# Tocolysis with nifedipine versus atosiban and perinatal outcome: an individual participant data meta-analysis

**DOI:** 10.1186/s12884-022-04854-1

**Published:** 2022-07-15

**Authors:** Tijn M. S. van Winden, Tobias A. J. Nijman, C. Emily Kleinrouweler, Raed Salim, Maryam Kashanian, Wafa R. Al-Omari, Eva Pajkrt, Ben W. Mol, Martijn A. Oudijk, Carolien Roos

**Affiliations:** 1 Department of Obstetrics, Amsterdam UMC location University of Amsterdam, Meibergdreef 9, Amsterdam, The Netherlands; 2 Amsterdam Reproduction and Development Research Institute, Amsterdam, The Netherlands; 3 Department of Obstetrics and Gynecology, Medisch Centrum Haaglanden, The Hague, Netherlands; 4 Department of Obstetrics and Gynecology, Emek Medical Center, Afula, Israel; 5 Department of Obstetrics & Gynecology, Akbarabadi Teaching Hospital, Tehran, Iran; 6 Department of Obstetrics and Gynecology, Medical City Teaching Hospital, Baghdad, Iraq; 7 Department of Obstetrics and Gynaecology, Monash University, Clayton, Victoria Australia; 8 Department of Obstetrics, Amsterdam UMC, location Vrije Universiteit Amsterdam, Boelelaan, 1117 Amsterdam, The Netherlands; 9 Department of Obstetrics and Gynaecology, Amsterdam Reproduction and Development Research Institute, Amsterdam UMC, Location AMC, H4-275, PO Box 22660, Amsterdam, 1100 DD the Netherlands

**Keywords:** Individual participant data meta-analysis, Obstetric labor, premature, Preterm Prelabor rupture of fetal membranes, PPROM, Tocolysis, Preterm birth, Preterm labor

## Abstract

**Background:**

Worldwide, nifedipine and atosiban are the two most commonly used tocolytic agents for the treatment of threatened preterm birth. The aim of this study was to evaluate the effectiveness of nifedipine and atosiban in an individual participant data meta-analysis (IPDMA).

**Methods:**

We investigated the occurrence of adverse neonatal outcomes in women with threatened preterm birth by performing an IPDMA, and sought to identify possible subgroups in which one treatment may be preferred. We searched PubMed, Embase, and Cochrane for trials comparing nifedipine and atosiban for treatment of threatened preterm birth between 24^0/7^ and 34^0/7^ weeks’ gestational age. Primary outcome was a composite of perinatal mortality and neonatal morbidities including respiratory distress syndrome, intraventricular haemorrhage, periventricular leucomalacia, necrotising enterocolitis, and sepsis. Secondary outcomes included NICU admission, prolongation of pregnancy and GA at delivery. For studies that did not have the original databases available, metadata was used. This led to a two-stage meta-analysis that combined individual participant data with aggregate metadata.

**Results:**

We detected four studies (*N* = 791 women), of which two provided individual participant data (*N* = 650 women). The composite neonatal outcome occurred in 58/364 (16%) after nifedipine versus 69/359 (19%) after atosiban (OR 0.76, 95%CI 0.47–1.23). Perinatal death occurred in 14/392 (3.6%) after nifedipine versus 7/380 (1.8%) after atosiban (OR 2.0, 95%CI 0.80–5.1).

Nifedipine results in longer prolongation of pregnancy, with a 18 days to delivery compared with 10 days for atosiban (HR 0.83 (96% CI 0.69–0.99)). NICU admission occurred less often after nifedipine (46%) than after atosiban (59%), (OR 0.32, 95%CI 0.14–0.75). The sensitivity analysis revealed no difference in prolongation of pregnancy for 48 hours (OR 1.0, 95% CI 0.73–1.4) or 7 days (OR 1.3, 95% CI 0.85–5.8) between nifedipine and atosiban. There was a non-significant higher neonatal mortality in the nifedipine-exposed group (OR 1.4, 95% CI 0.60–3.4).

**Conclusions:**

In this IPDMA, we found no differences in composite outcome between nifedipine and atosiban in the treatment of threatened preterm birth. However, the non-significant higher mortality after administering nifedipine warrants further investigation of the use of nifedipine as a tocolytic drug.

**Study registration:**

We conducted this study according to a prospectively prepared protocol, registered with PROSPERO (the International Prospective Register of Systematic Reviews) under CRD42016024244.

**Supplementary Information:**

The online version contains supplementary material available at 10.1186/s12884-022-04854-1.

## Tweetable abstract

IPDMA of two RCTs comparing tocolytics nifedipine and atosiban as treatment of threatened preterm birth. Baby outcomes were similar. Although mortality was higher with nifedipine, this difference could have occurred by chance.

## Key message

In this comparison between nifedipine and atosiban, no drug is clearly superior to the other. Nifedipine is associated with a prolongation of pregnancy. However, this does not yield better neonatal outcomes.

## Background

Preterm birth is one of the biggest challenges in obstetrics, responsible for an estimated 1.12 million neonatal deaths per year [1, 2]. Preterm birth is the largest cause of neonatal death and the second leading cause of deaths among children under the age of 5 years, making it one of the main direct causes of infant mortality [3].

An important intervention to improve neonatal outcome is administration of antenatal corticosteroids to enhance fetal lung maturation in case of threatened preterm birth. Antenatal corticosteroids require 48 hours to reach the maximum effect. Because of this, many protocols have incorporated administration of tocolytic drugs to delay birth for 48 hours [4]. When applicable to the local situation, in utero transfer to a tertiary center is crucial.

In the past decades, various tocolytic agents have been investigated and made available for clinical use [4]. Currently, the two most commonly used tocolytic drugs are atosiban, an oxytocin receptor antagonist, and nifedipine, a calcium channel blocking agent. However, there is no consensus on which drug is the optimal drug to use in clinical practice, also regarding safety. The heterogeneity in treatment strategies and outcome measures have made it difficult to interpret study outcomes in a clinical setting. We believe that perinatal outcome should be the main treatment goal, more important than prolongation of pregnancy. With this study, we aim to address this knowledge gap. Moreover, in specific subsets of women with threatened preterm birth, such as with ruptured membranes versus intact membranes the optimal drug might differ from the general population with threatened preterm birth.

## Methods

### Aim

To systematically asses the question which is the optimal tocolytic agent to administer, we performed an individual participant data meta-analysis (IPDMA) in which we compared nifedipine with atosiban with respect to neonatal outcome in women with threatened preterm birth and multiple subgroups.

### Trial registration

This IPDMA was conducted according to a prospectively prepared protocol, registered with PROSPERO (the International Prospective Register of Systematic Reviews) under CRD42016024244. We followed the Preferred Reporting Items for Systematic reviews and Meta-analyses (PRISMA) guidelines for the meta-analysis of randomized controlled trials [5].

### Sources and search strategy

We searched PubMed, Embase and Cochrane databases for trials that compared tocolysis with nifedipine and atosiban in threatened preterm birth. We used the keywords and synonyms of “nifedipine” AND “atosiban” AND “preterm birth”. Furthermore, we searched clinicaltrials.gov and isrctn.com for continuing or unpublished studies. The last search was conducted on 13 April 2022. The search strategy is presented in appendix S1.

### Eligibility criteria and study selection

Studies were eligible for inclusion in this IPDMA if they were a randomized controlled trial comparing nifedipine with atosiban for 48 hours of tocolysis in women with threatened preterm birth between 24^0/7^ and 34^0/7^ weeks of gestation.

Corresponding authors of eligible studies were contacted and invited to participate in the IPDMA. Authors who replied positively were provided with a detailed project protocol and they were asked to send their original, anonymized data. Additional information was extracted from the original papers. Shared data were reformatted or recoded if necessary to acquire uniformity, and merged into one database.

### Outcomes

The primary outcome was a composite of neonatal morbidities and perinatal mortality, according to the registered study protocol, where available. Neonatal morbidities included bronchopulmonary dysplasia (BPD), periventricular leucomalacia (PVL) ≥ stage II, intraventricular hemorrhage (IVH) ≥ grade III, necrotizing enterocolitis (NEC) ≥ stage II and culture proven sepsis.

Secondary outcomes were time to delivery, gestational age at delivery, successful 48 hours of tocolysis, maternal side effects, blood loss during delivery and days on ventilation support.

As not all planned outcome measures were available in the shared datasets, our composite outcome was as follows: respiratory distress syndrome (RDS, requiring surfactant), periventricular leucomalacia (PVL) ≥ stage II, intraventricular hemorrhage (IVH) ≥ grade III, necrotizing enterocolitis (NEC) ≥ stage II and culture proven sepsis.

Additional available outcomes where: NICU admission, ventilation support, total days in hospital, total days in hospital until corrected age 3 months, apnea and birthweight.

### Statistical analyses

Data on baseline characteristics and outcomes were summarized for the complete database and for the two study groups within each study. Continuous variables were presented as means with standard deviations (SD) or as medians with interquartile ranges (IQR), as appropriate. Dichotomous or categorical variables were presented as the number and percentage of the study-specific or total study population. Outcomes were analyzed by an intention-to-treat approach. For binomial outcomes, we used a mixed model with a log link, thus resulting in odds ratios (OR) with 95% confidence intervals (95% CI). These models include a random intercept to account for differences in prevalence between studies and to control for between-study heterogeneity [6]. Gestational age at delivery and time to delivery were analyzed with Cox proportional hazard models, stratified at the study level, and Kaplan-Meier estimates and tested with a log-rank test. We censored gestational age at delivery at 37^+0^ weeks of gestation as we were mainly interested in the effect of tocolytic therapy on prolonging pregnancy in the preterm period.

For outcomes on child level, we accounted for interdependence between outcomes of babies of the same mother in multiple pregnancies [7]. We assessed binary outcomes with a generalized estimating equations (GEE) model for binomial data with an unstructured correlation matrix, considering the mother as a cluster variable. Odds ratios with 95% CI and P values are reported. Likewise, we evaluated continuous outcomes on the child level with linear quantile mixed models with the mother as a grouping variable, resulting in a median difference with 95% CI [8].

The presence of statistical heterogeneity of outcomes across studies was assessed using the I^2^ measure and interpreted as follows: 0% indicated no observed heterogeneity; 25, 50, and 75% indicate low, moderate, and high heterogeneity, respectively [9].

Data preparation and statistical analyses were performed using SPSS (version 25.0, IBM Corp, Armonk, NY, USA) and R (version 3.5.1, R Core Team, Vienna, Austria).

### Risk of bias assessment

Two review authors independently assessed the risk of bias of each included study against key criteria: random sequence generation; allocation concealment; blinding of participants, personnel and outcomes; incomplete outcome data; selective outcome reporting; and other sources of bias, in accordance with methods recommended by The Cochrane Collaboration [10]. The following judgements were used: low risk, high risk, or unclear (either lack of information or uncertainty over the potential for bias). Authors resolved disagreements by consensus, and a third author was consulted to resolve disagreements if necessary.

### Subgroup analysis

We investigated possible subgroup effects for women with ruptured and intact membranes, gestational age at randomization <30^+0^ versus ≥30^+0^ weeks, cervical dilatation at randomization <2 versus ≥2 cm, singleton and multiple pregnancies, nulliparous and parous women, women with and without a history of preterm birth, women with and without a history of term birth, and neonatal sex (boys versus girls).

Subgroup effects were studied by including an interaction term between the subgrouping variable and treatment allocation in the regression model. When the interaction term was found to be significant (*P* < 0.10), we performed a stratified analysis in different strata of the subgroups. Subgroup analyses were only performed for gestational age at delivery, time to delivery, successful 48 hours of tocolysis, composite adverse neonatal outcome and ventilation support.

### Sensitivity analysis

To aggregate all possible evidence, including studies not providing individual participant data, we performed a sensitivity analysis. We combined individual participant data when available, and aggregate metadata for studies who did not provide individual patient data in a two-stage meta-analysis.

## Results

### Study selection

The literature search resulted in 176 citations, showing 4 trials that were eligible for inclusion, Van Vliet et al., Lancet 2016; Salim et al., Obstet Gynecol 2012; Al-Omari et al., Eur J Obstet Gynecol Reprod Biol 2006; Kashanian et al., Int J Gynecol Obstet 2005 [11–14]. A search on clinicaltrials.gov and isrctn.com resulted in no additional suitable trials. Details on study selection and the search strategy are specified in Appendix S1. 

All four corresponding authors were interested in collaboration. From two studies (Al-Omari et al. and Kashanian et al.), individual participant data could not be provided because it was reported that the original database was lost [13, 14]. Thus, from the four studies reporting on 791 women, we could use individual participant data of the two largest studies (*n* = 650 women). The original databases were used to construct the IPDMA data set. As the shared datasets had either (almost) complete data for all variables or additional variables were not recorded at all, we did not impute missing data. Additional investigation based on all studies’ metadata was conducted as sensitivity analysis.

### Study characteristics

Sample sizes of the studies were 505 and 145 women; 324 women were treated with nifedipine and 326 women with atosiban as tocolytic therapy. All studies evaluated nifedipine treatment against atosiban treatment [11–14].

### Results of IPDMA (synthesis of two largest studies)

Table 1 shows the baseline characteristics of the nifedipine and atosiban groups for all included women and in the two separate trials. Most characteristics were distributed evenly, indicating no clinical difference between the nifedipine and atosiban group. There were slightly more multiples in the nifedipine group (22.3%) than in the atosiban group (18.1%).

**Table 1:** Baseline characteristics

Characteristics	IPDMA		Van Vliet		Salim	
	Nifedipine (*n* = 324)	Atosiban (*n* = 326)	Nifedipine (*n* = 249)	Atosiban (*n* = 256)	Nifedipine (*n* = 75)	Atosiban (*n* = 70)
Age (years)	30.1 (26.0–34.0)	30.0 (26.6–33.0)	30.7 (26.2–34.1)	30.2 (27.1–33.1)	27.0 (25.0–33.0)	28.0 (25.0–32.3)
BMI (kg/m^2^) ^a^	22.7 (20.4–25.6)	22.8 (20.4–25.5)	23.1 (20.8–25.8)	22.8 (20.5–25.6)	21.4 (19.9–25.0)	22.6 (20.2–25.3)
Nulliparous	197/323 (61)	195/325 (60)	160/248 (65)	170/255 (67)	37 (49)	25 (36)
Multiple pregnancy ^b^						
- Twin	72 (22)	58 (18)	49 (20)	37 (15)	23 (31)	21 (30)
- Triplet	0	1 (0.3)	0	1 (0.4)	0	0
Previous preterm birth	45/323 (14)	45/324 (14)	33/248 (13)	30/254 (12)	12 (16)	15 (21)
Gestational age at study entry (weeks) ^b^	31.1 (29.0–33.0)	30.8 (28.9–32.6)	30.9 (28.7–32.9)	30.7 (28.6–32.4)	31.9 (30.0–33.1)	31.1 (29.7–32.8)
PPROM at study entry ^b^	85 (26)	88 (27)	85 (34)	88 (34)	0	0
Vaginal examination at study entry						
- Dilatation (cm) ^c^	1.0 (1.0–2.0)	1.5 (1.0–2.0)	1.0 (1.0–2.0)	1.0 (1.0–2.0)	1.5 (1.0–2.0)	1.5 (1.0–2.0)
- Effacement (%) ^d^	75 (50–100)	75 (50–100)	75 (50–100)	75 (50–100)	75 (50–75)	75 (50–75)

Data are median (IQR), n(%) or n/N (%). *BMI* Body mass index

^a^nifedipine *n* = 273 (198 + 75), atosiban *n* = 277 (207 + 70)

^b^nifedipine *n* = 323 (248 + 75), atosiban *n* = 326 (256 + 70)

^c^nifedipine *n* = 187 (112 + 75), atosiban *n* = 191 (121 + 70)

^d^nifedipine *n* = 162 (87 + 75), atosiban *n* = 170 (100 + 70)

Women included in Van Vliet et al. were slightly older, had a higher BMI and lower gestational age at randomization, compared to Salim et al. In Salim’s population, more women had a twin pregnancy and more women experienced previous preterm birth.

The composite outcome did not differ significantly between the nifedipine and the atosiban group (15.9% versus 19.2% respectively, OR 0.76, 95% CI 0.47–1.23). Similarly, there was no significant difference in perinatal death (3.6% versus 1.8%, OR 2.01, 95% CI 0.80–5.06). All components of the primary outcome are listed in Table 2 and secondary outcomes in Table 3.

**Table 2:** Primary outcome and composites

Characteristics	IPDMA			Van Vliet			Salim		
	Nifedipine (*n* = 392)	Atosiban (*n* = 380)	OR or MD (95% CI)	Nifedipine (*n* = 294)	Atosiban (*n* = 289)	OR or MD (95% CI)	Nifedipine (*n* = 98)	Atosiban (*n* = 91)	OR or MD (95% CI)
Composite outcome^a^	58/364 (16)	69/359 (19)	0.76 (0.47–1.2), *p* = 0.27	51/267 (19)	60/268 (22)	0.77 (0.12–5.0), *p* = 0.79	7/97 (7.2)	9 (9.9)	0.63 (0.017–24), *p* = 0.81
Perinatal death	14 (3.6)	7 (1.8)	2.01 (0.80–5.1), *p* = 0.14	14 (4.8)	7 (2.4)	2.01 (0.80–5.1), *p* = 0.14	0	0	–
Intraventricular hemorrhage	6/391 (1.5)	3/379 (0.8)	1.95 (0.49–7.9), *p* = 0.35	5 (1.7)	2/288 (0.70)	2.47 (0.48–13), *p* = 0.28	1/97 (1.0)	1 (1.1)	0.94 (0.058–15), *p* = 0.96
Necrotizing enterocolitis	7 (1.8)	8/379 (2.1)	0.84 (0.30–2.4), *p* = 0.74	7 (2.4)	4/288 (1.4)	1.73 (0.50–6.0), *p* = 0.39	0	4 (4.4)	–
Respiratory distress syndrome	37 (10)	44 (12)	0.76 (0.090–6.4), *p* = 0.80	32/267 (12)	41/267 (15)	0.72 (0.070–7.4), *p* = 0.79	5 (5.1)	3 (3.3)	1.39 (0.008–242), *p* = 0.90
Culture-proven sepsis	27 (6.9)	27/379 (7.1)	0.87 (0.058–13), *p* = 0.92	24 (8.2)	25/288 (8.7)	0.84 (0.051–14), *p* = 0.91	3 (3.1)	2 (2.2)	1.34 (0.00–1566), *p* = 0.95

Data are median (IQR) or n/N (%). OR: odds ratio. MD: median difference

^a^includes perinatal death, IVH, NEC, RDS requiring surfactant and culture-proven sepsis

**Table 3:** Neonatal and maternal secondary outcomes

Characteristics	IPDMA			Van Vliet			Salim		
Maternal outcomes	Nifedipine (***n*** = 321)	Atosiban (***n*** = 321)	HR or OR (95% CI) or χ^**2**^ test	Nifedipine (***n*** = 246)	Atosiban (***n*** = 251)	HR (95% CI) or χ^**2**^ test	Nifedipine (***n*** = 75)	Atosiban (***n*** = 70)	HR (95% CI) or χ^**2**^ test
**GA at delivery (weeks)**^a^	33 + 6 (31 + 1–37 + 2)	33 + 0 (30 + 3–36 + 3)	HR 0.80 (0.67–0.95), *p* = 0.013	33 + 1 (30 + 4–37 + 0)	32 + 3 (30 + 1–36 + 0)	HR 0.85 (0.70–1.03), *p* = 0.10	36 + 4 (34 + 0–38 + 1)	34 + 5 (33 + 0–38 + 0)	HR 0.60 (0.40–0.90), *p* = 0.014
**Time to delivery (days)**^a^	18 (2–44)	10 (2–42)	HR 0.83 (0.69–0.99), *p* = 0.038	7.5 (1.0–39.0)	4.0 (1.0–38.5)	HR 0.88 (0.72–1.06), *p* = 0.18	35.0 (23.0–49.5)	32.0 (15.0–50.0)	HR 0.61 (0.40–0.94), *p* = 0.024
**Successful 48 hours of tocolysis**	247 (77)	243 (76)	OR 1.06 (0.73–1.53), *p* = 0.77	177 (72)	181 (72)	OR 0.99 (0.67–1.47)	70 (93)	62 (89)	OR 1.81 (0.56–5.81)
**Neonatal outcomes**	**Nifedipine (*n*** **= 392)**	**Atosiban (*n*** **= 380)**	**OR or MD (95% CI)**	**Nifedipine (*n*** **= 294)**	**Atosiban (*n*** **= 289)**	**OR or MD (95% CI)**	**Nifedipine (*n*** **= 98)**	**Atosiban (*n*** **= 91)**	**OR or MD (95% CI)**
**NICU admission**	182 (46)	225 (59)	0.32 (0.14–0.75), *p* = 0.008	153 (52)	179 (62)	0.53 (0.29–0.96), *p* = 0.037	29 (30)	46 (51)	0.32 (0.018–5.71), *p* = 0.44
**Ventilation support**									
**- CPAP**	–	–	–	111/251 (44)	131/252 (52)	0.69 (0.44–1.07), *p* = 0.098	–	–	0
**- Intubation**	52/355 (15)	66/349 (19)	0.65 (0.13–3.38), *p* = 0.61	42/257 (16)	53/258 (21)	0.68 (0.098–4.73), *p* = 0.70	10 (10)	13 (14)	0.56 (0.033–9.71), *p* = 0.69
**Birthweight**	2077 (1604–2717)	1985 (1532–2568)	112 (−85–309), *p* = 0.26	1983 (1503–2674)	1845 (1435–2465)	139 (−59–336), *p* = 0.16	2351 (1985–2894)	2243 (1930–2761)	251 (−47–550), *p* = 0.097

Data are median (IQR) or n/N (%). *OR* Odds ratio, *MD* Median difference

^a^censored at 37 + 0 weeks

^b^includes perinatal death, sepsis, IVH, NEC, RDS

**Fig. 1:**
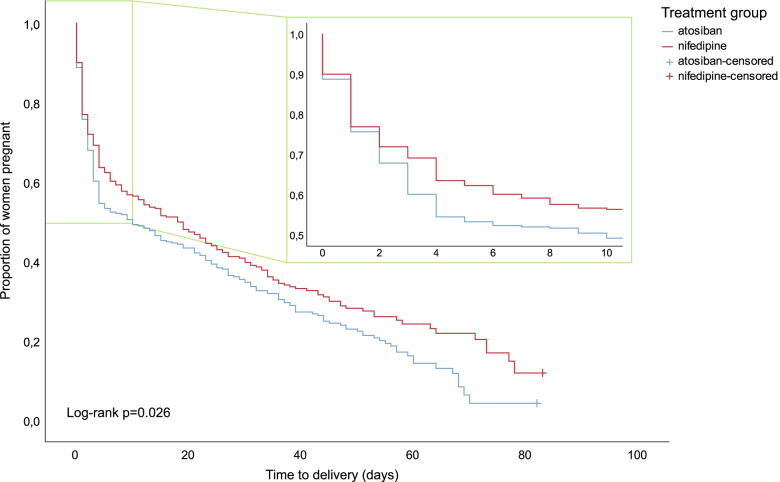
Prolongation of pregnancy, censored at 37 weeks

The Kaplan–Meier curve for prolongation of pregnancy (Fig. 1) demonstrated a log-rank test with P = 0.026, indicating that nifedipine results in longer prolongation of pregnancy, regardless of the gestational age at study inclusion. This corresponds with a longer time to delivery of 18 days with nifedipine versus 10 days with atosiban (HR 0.83, 95% CI 0.69–0.99, P = 0.038) and a higher gestational age at delivery of 33^+6^ week with nifedipine versus 33^+0^ weeks with atosiban (HR 0.80, 95% CI 0.67–0.95, P = 0.013). None of the other secondary outcomes, such as IVH, NEC, RDS or sepsis occurred more frequently in one of the treatment groups.

### Subgroup analysis

Subgroup analyses of the effect of treatment allocation are summarized in supplementary Appendix S2. Regarding the planned subgroup analysis with stratification by cervical length less than 15 mm, the trial of Salim et al. did not register cervical length data. Otherwise, the data available from the trials were eligible for comparison.

Nifedipine was associated with a statistically significant increase in gestational age at delivery and time to delivery in women with intact membranes (OR 0.70 (95% CI 0.56–0.87) and 0.71 (95% CI 0.57–0.89), respectively). Successful tocolysis for at least 48 hours was achieved with nifedipine more often in subgroup of more than 2 cm dilatation (OR 2.2 (95% CI 1.07–4.76)). Moreover, for women with a history of preterm birth, nifedipine-exposed children less often faced the composite adverse outcome (OR 0.18 (95% CI 0.034–0.92)). This effect was not visible in women with no history of preterm birth or in women with or without a history of term birth.

The composite adverse neonatal outcome did not differ significantly when comparing women with or without ruptured membranes, with gestational age at study entry less or above 30 weeks, with dilatation at study entry less or above 2 cm, when comparing singleton and multiple pregnancies or nulliparous and multiparous women. Lastly, no significant difference in composite adverse outcome could be related to neonatal sex.

### Sensitivity analysis (synthesis of four studies, using individual data when available)

We performed a sensitivity analysis by pooling the reported data of all four studies that directly compared neonatal outcomes for tocolysis using nifedipine and atosiban in an aggregate meta-analysis.

There was no difference in prolongation of pregnancy for 48 hours (OR 1.0, 95% CI 0.73–1.4) or 7 days (OR 1.3, 95% CI 0.85–5.8) between nifedipine and atosiban. However, when looking at mortality, there is a non-significant higher neonatal mortality in the nifedipine-exposed group (OR 1.4, 95% CI 0.60–3.4). These results are visualized in Appendix S3.

### Risk of bias assessment

The risk of bias of the included studies was assessed independently by two reviewers (TN and CR) using the Cochrane Collaboration’s risk-of-bias tool, as visualized in Table 4 [10]. This includes the assessment of adequate sequence generation, allocation concealment, blinding of participants and personnel, and blinding for outcome measures, incomplete outcome data, and other sources of bias. Both of the studies were considered to be at low risk of bias arising from the randomization process, bias due to missing outcome data and bias in measurement of the outcome. Selective reporting of outcome was not considered as a relevant bias in this IPDMA, as original databases are used instead of reported outcomes. For both studies the assessors reported some concerns regarding bias due to deviations from intended intervention. The leading cause for this, is the tool’s subheading “were participants aware of their assigned intervention during the trial?”. Due to the obligatory routes of administration of the used tocolytics, complete blinding of the group allocation was not possible: nifedipine is given orally, while atosiban is solely administered intravenously. This leads the overall risk of bias score for both studies to be “some concerns”.

**Table 4:** Risk of bias in included studies

Study	Random sequence generation	Deviations from intended intervention	Missing outcome data	Measurement of the outcome	Selection of reported results	Overall
**Salim 2012** [12]						
**Van Vliet 2016** [11]						

Risk of bias judgement  Low risk of bias  Some concerns  High risk of bias

## Discussion

In this IPDMA encompassing 650 women, no clear superior tocolytic could be determined. Prolongation of pregnancy was longer with nifedipine, independent of gestational age. This was also reflected in a longer time to delivery and higher gestational age at delivery. However, the prolonged pregnancy did not reduce the rate of adverse neonatal outcome, apart from a lower rate of NICU admission with nifedipine. A sensitivity analysis including all RCT’s that compared nifedipine and atosiban did not show a prolongation of pregnancy for 48 hours or 7 days. We found a non-significant higher number of neonatal deaths in the nifedipine group. These findings should be taken into account when considering atosiban or nifedipine in women presenting with a threatened preterm birth.

### Strengths and limitations

This study has several strengths: by performing an IPDMA, we could investigate a relatively large population and this allowed for more power in subgroup analyses. Second, by analyzing individual participant data, we could overcome the problem of selective reporting. This also resulted in some outcomes in this IPDMA differing from the reported outcomes in the original articles. For example, in our analyses we only considered a neonate to have RDS when the clinical condition was severe enough to warrant administration of surfactant, while this differed from one of the included studies. Another explanation is the exclusion of women who did not meet the criteria defined in our study protocol. This was the case for 6 women in the database of Van Vliet et al., leading to exclusion of two cases of perinatal mortality.

Lastly, in rare cases the new analyses for this IPDMA resulted in minor differences from the published data.

It should be noted that the included studies levelled out some of their reported outcomes such as neonatal morbidities which in the study by Van Vliet et al. were more frequent with atosiban and in Salim et al. occurred more often with nifedipine.

Our study also has some limitations. As not all of the planned outcome measures were available, it was not feasible to perform an IPDMA with our initial planned data points. Primarily we chose to implement the outcomes as defined in the Core Outcome Set published in 2016 [15]. Since Salim’s study was published before this in 2012, not all of the outcomes were reported. On the other hand, maternal side effects were reported in different items e.g., headache, palpitations, nausea, pruritus, but were not accurately recorded in Van Vliet et al. We had to redefine one of our IPDMA outcome measures accordingly and sought to find adequate proxies for initial outcome measures as planned in protocol. As measure for airway and breathing problems, we planned to include BPD. This was not reported in the study of Salim, but RDS was recorded in both databases, so we chose to report this instead, preventing excluding outcomes on breathing problems entirely. On the other hand, we could include additional outcomes such as NICU admission, ventilation support, total days in hospital, total days in hospital until corrected age 3 months, apnea and birthweight, that were recorded in both databases.

Unfortunately, we could not retrieve the entire outcome databases of two other studies, and it was thus not possible to perform an IPDMA on all the RCT’s comparing nifedipine and atosiban. In addition, these studies did not assess all outcomes predefined in the protocol.

One point deserving attention, is a possible difference in risk profile between women in the two studies. Women included in Van Vliet et al. are slightly older, have a higher BMI and lower gestational age at randomization, compared to Salim et al. Moreover, this trial included women with ruptured membranes, which in itself drastically increases the risk to deliver. Given that we wanted to include all available evidence in a field of tocolytics where high-quality trials are already scarce, also women with PPROM were included. Many guidelines from societies across the world advocate administering tocolytics, also in case of ruptured membranes in absence of clinical infection. Because there is a chance that the outcome may be different between women with and without ruptured membranes, we performed a subgroup analysis for women with ruptured membranes versus intact membranes. This yielded no different outcome between these groups.

Women with PPROM in the Apostel III RCT were treated according to the national guideline “Threatened preterm birth” by the NVOG (Dutch Society of Obstetrics and Gynaecology) [16]. This guideline indicates administration of corticosteroids and tocolytics for 48 hours for women in threatened preterm birth between 24 and 34 weeks of gestation. Afterwards, if no signs of infection are present, expectative management is advocated. Preventive treatment with antibiotics in women with PPROM was not routinely initiated, unless a patient had been diagnosed as a GBS carrier or if there were clinical signs of intra-uterine infection. In this case, treatment was initiated according to local protocol. From the women in the latter group on the other hand, more carried a twin pregnancy and more women experienced previous preterm birth. Of course, the question remains whether this possible heterogenicity should be translated in an implication for treatment choice. Since we compared differences in outcomes between nifedipine and atosiban and those groups are evenly represented in both databases, we believe the combined database offers a picture representative for patients in daily practice.

### Interpretation and clinical implications

The apparent superiority for nifedipine regarding prolongation of pregnancy that was described in the trial of Salim et al. and that was visualized in the survival curve of Van Vliet et al., is a promising sign that was captured in Fig. 1. However, this advantage did not lead to a better neonatal outcome. Neither could a difference be demonstrated by pooling this data with all other available evidence, as shown in the sensitivity analysis. The lower rate of NICU admission we found in the nifedipine-exposed group, may be attributed to the higher gestational age at delivery. However, as noted, this did not lead to the generally accepted consequence that this also improves neonatal outcome as this would have been reflected in reduction in perinatal mortality, RDS, IVH, PVL, NEC or sepsis.

Another possible explanation can be derived from a follow-up study, where mortality until the age of 2.5–5.5 years was higher in the nifedipine group, while for the remaining group healthy survival occurred more often than in atosiban-exposed children [17].

Based on this finding, the hypothesis was raised that there seems to be a trade-off between these two outcomes, possibly suggesting that the non-significantly higher mortality with nifedipine, also leaves the surviving children on average less impacted by preterm birth; the same mechanism could contribute to the demonstrated lower rate of NICU admittance. However, thorough research with a large number of participants should be conducted to answer this question.

Summarized, a slightly longer delay of delivery with nifedipine did not reflect in better neonatal outcomes. The non-significant higher mortality rate in the nifedipine group remains a matter of concern. While nifedipine is a more potent tocolytic agent, in certain subgroups of patients with threatened preterm birth, prolongation of pregnancy may not be beneficial for the infant. Another explanation is that the surviving part of neonates exposed to nifedipine were relatively less impaired than their counterparts in the atosiban group. Although changes in uterine blood flow, occurrence of fetal acidemia and reduced fetal movements have been demonstrated in animals, neither of these effects was observed in human studies [18–25].

The field of tocolytics, although studied for a long time, remains to be surrounded by many questions. In this respect, it is disappointing that only four studies reported on tocolysis with nifedipine and atosiban in threatened preterm birth. Since we believe that individual participant data is the preferred source for performing a meta-analysis, we stuck to the studies that provided these, hence reducing the number of serviceable studies to two. Without question, it would be desirable to collect more data in order to draw more definitive conclusions.

The paucity of literature describing (long-term) neonatal outcomes, raises a problem for clinicians considering a tocolytic for their patients. The optimal drug of choice should be not only effective in delaying delivery, but should improve neonatal outcome, as compared to placebo or no treatment. In addition, these drugs should have a favorable side effect profile on both maternal and fetal side. Also, long term follow-up of these studies should be performed in order to assess the effect on the child later in life. Placebo-controlled trials, especially on the currently used drugs, are rare, and not of sufficient size to draw conclusions. Therefore, we urge clinicians to re-evaluate the policy regarding the use of tocolysis. It is our opinion that tocolytic drugs should only be used within the setting of a placebo-controlled trial, with neonatal outcome as a primary outcome, and assessing long term follow-up. The WHO guideline on preterm birth is in agreement with this statement and does not advice to use tocolytics for the purpose of improving neonatal outcomes [26].

## Conclusion

Nifedipine and atosiban are equally effective in the treatment of threatened preterm birth. Nifedipine results in a lower rate of NICU admission and longer duration of pregnancy, however did not result in an improved neonatal outcome. In women with a history of preterm birth, the composite outcome occurred less frequently in nifedipine-exposed children. There is a non-significant higher mortality after administering nifedipine which warrants further investigation of the use of nifedipine as a tocolytic drug.

## Supplementary Information

**Additional file 1.**

**Additional file 2.**

## Data Availability

The datasets used and/or analysed during the current study are available from the corresponding author on reasonable request.

## Abbreviations

IPDMA: Individual participant data meta-analysis
PPROM: Preterm prelabor rupture of membranes
