# Supplementary material for: Tocolysis with nifedipine versus atosiban and perinatal outcome: an individual participant data meta-analysis
Source: BMC Pregnancy Childbirth. 2022 Jul 15;22:567. doi: 10.1186/s12884-022-04854-1 (PMC9284745; doi:10.1186/s12884-022-04854-1)
Supplement: Supplementary file 1 — Additional file 1. [file 12884_2022_4854_MOESM1_ESM.docx]

**Appendix S1: flow chart search strategy**


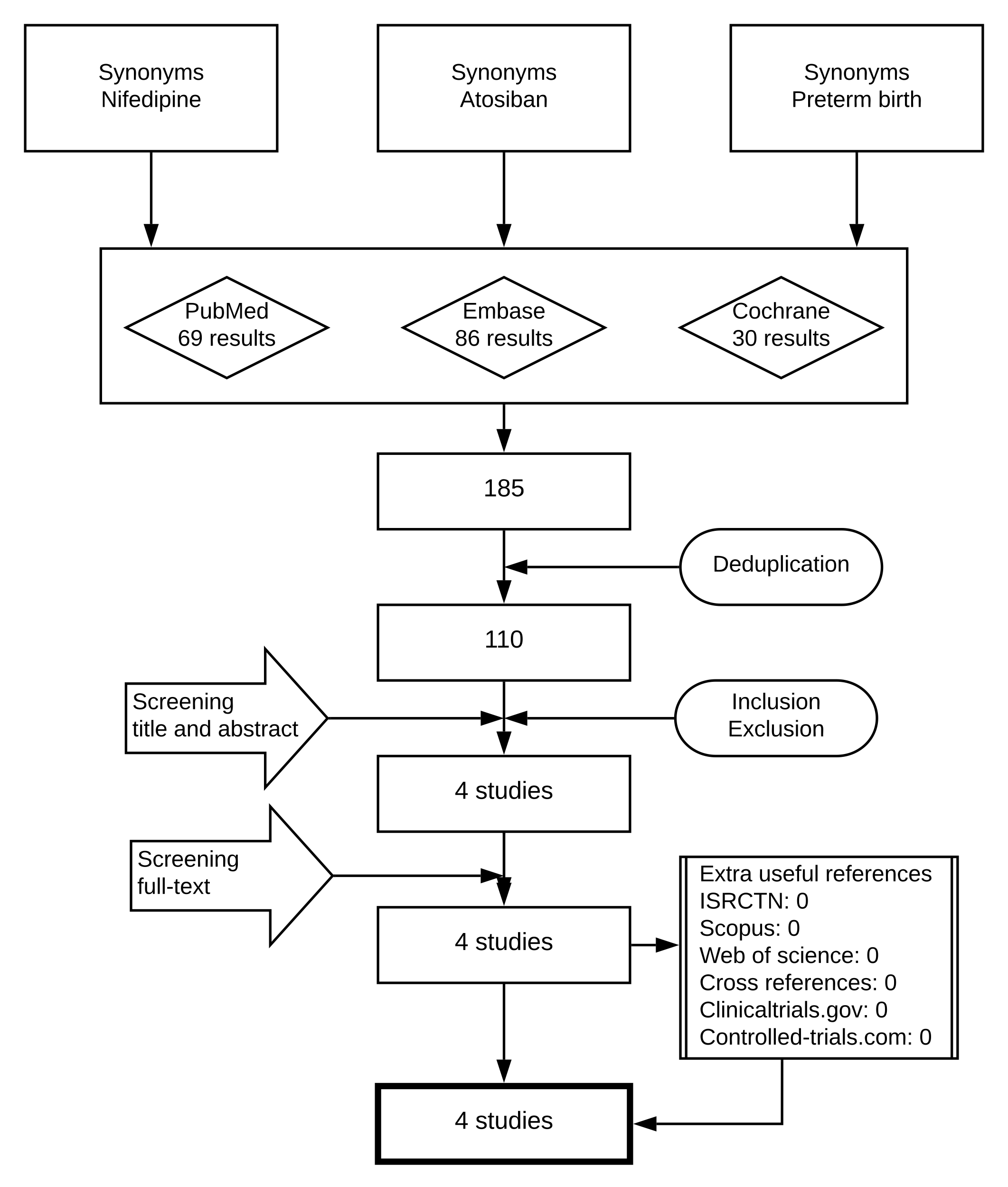


**Search terms**

Pubmed 13 April 2022: 69 results

("nifedipine"[MeSH Terms] OR "nifedipine"[All Fields]) AND ("atosiban"[Supplementary Concept] OR "atosiban"[All Fields]) AND ("obstetric labor, premature"[MeSH Terms] OR "preterm birth"[All Fields] OR "premature birth"[All Fields] OR "preterm labor"[All Fields] OR "premature labor"[All Fields] OR "preterm labour"[All Fields] OR "premature labour"[All Fields])

Cochrane Central Register of Controlled Trials 13 April 2022: 30 results

Nifedipine AND atosiban (Title Abstract Keyword, including word variations) in Trials

Embase 13 April 2022: 86 results

nifedipine:ab,ti AND atosiban:ab,ti AND (premature:ab,ti OR preterm:ab,ti)

**Appendix S2: Subgroup analyses**

| **Ruptured vs intact membranes** | ***P* interaction term** | **Nifedipine** | **Atosiban** | **OR (95% CI)** | **Nifedipine** | **Atosiban** | **OR (95% CI)** |
| --- | --- | --- | --- | --- | --- | --- | --- |
| **Maternal outcomes** |  | **Ruptured membranes (n=171)** | | | **Intact membranes (n=470)** | | |
| GA at delivery (weeks) | 0.015 | 31+6 (30+1-33+3) | 32+1 (30+2-33+4) | HR 1.11 (0.82-1.20), p=0.52 | 35+4 (31+6-38+0) | 34+0 (30+4-37+4) | 0.70 (0.56-0.87), p=0.0016 |
| Time to delivery (days) | 0.0031 | 2 (0-6) | 2 (1-6) | HR 1.18 (0.87-1.630), p=0.29 | 31 (6-53) | 24 (2-51) | 0.71 (0.57-0.89), p=0.003 |
| Successful 48 hours of tocolysis | 0.11 | 46/85 (54) | 53/86 (62) |  | 201/235 (86) | 190/235 (81) |  |
| **Neonatal outcomes** |  | **Ruptured membranes (n=204)** | | | **Intact membranes (n=567)** | | |
| Composite outcome | 0.77 | 17/99 (17.2) | 23/102 (22.5) |  | 40/264 (15.2) | 46/257 (17.9) |  |
| Ventilation support (intubation) | 0.91 | 14/95 (14.7) | 19/99 (19.2) |  | 37/259 (14.3) | 47/250 (18.8) |  |
| **Gestational age at study entry < 30 vs ≥ 30 weeks** | ***P* interaction term** | **Nifedipine** | **Atosiban** | **OR (95% CI)** | **Nifedipine** | **Atosiban** | **OR (95% CI)** |
| **Maternal outcomes** |  | **< 30 weeks (n=267)** | | | **≥ 30 weeks (n=375)** | | |
| GA at delivery (weeks) | 0.32 | 30+6 (28+6-37+0) | 31+0 (28+0-36+3) |  | 34+2 (32+6-37+5) | 33+4 (31+5-36+2) |  |
| Time to delivery (days) | 0.73 | 18 (2-59) | 23 (2-58) |  | 19 (2-38) | 6 (1-32) |  |
| Successful 48 hours of tocolysis | 0.32 | 102/128 (79.7) | 115/139 (82.7) |  | 145/193 (75.1) | 128/182 (70.3) |  |
| **Neonatal outcomes** |  | **< 30 weeks (n=325)** | | | **≥ 30 weeks (n=447)** | | |
| Composite outcome | 0.22 | 46/146 (31.5) | 49/155 (31.6) |  | 12/218 (5.5) | 20/204 (9.8) |  |
| Ventilation support (intubation) | 0.82 failed to converge | 38/144 (26.4) | 43/149 (28.9) |  | 14/211 (6.6) | 23/200 (11.5) |  |
| **Dilatation at study entry < 2 vs ≥ 2 cm** | ***P* interaction term** | **Nifedipine** | **Atosiban** | **OR (95% CI)** | **Nifedipine** | **Atosiban** | **OR (95% CI)** |
| **Maternal outcomes** |  | **< 2 cm (n=228)** | | | **≥ 2cm (n=147)** | | |
| GA at delivery (weeks) | 0.78 inner loop failed to converge | 35+5 (32+6-38+0) | 34+0 (31+1-37+0) |  | 34+0 (31+2-37+1) | 32+2 (30+1-35+5) |  |
| Time to delivery (days) | 0.93 | 18 (2-34) | 3 (1-34) |  | 34 (3-54) | 27 (3-51) |  |
| Successful 48 hours of tocolysis | 0.046 | 97/117 (82.9) | 95/111 (85.6) | 0.78 (0.38-1.62), p=0.51 | 55/70 (78.6) | 48/77 (62.3) | 2.26 (1.07-4.76), p=0.033 |
| **Neonatal outcomes** |  | **< 2 cm (n=272)** | | | **≥ 2cm (n=186)** | | |
| Composite outcome | 0.87 | 11/129 (8.5) | 15/124 (12.1) |  | 16/89 (18.0) | 24/88 (27.3) |  |
| Ventilation support (intubation) | 0.72 | 15/125 (12.0) | 19/120 (15.8) |  | 12/86 (14.0) | 24/88 (27.3) |  |
| **Singleton vs multiple pregnancy** | ***P* interaction term** | **Nifedipine** | **Atosiban** | **OR (95% CI)** | **Nifedipine** | **Atosiban** | **OR (95% CI)** |
| **Maternal outcomes** |  | **Singletons (n=513)** | | | **Multiples (n=129)** | | |
| GA at delivery (weeks) | 0.21 | 33+6 (31+1-38.0) | 33+3 (30+4-37+0) |  | 33+3 (31+2-35+3) | 31+3 (30+1-34+1) |  |
| Time to delivery (days) | 0.72 | 19 (2-48) | 10 (2-47) |  | 15 (2-34) | 9 (1-31) |  |
| Successful 48 hours of tocolysis | 0.63 | 193/250 (77.2) | 201/263 (76.4) |  | 54/71 (76.1) | 42/58 (72.4) |  |
| **Neonatal outcomes** |  | **Singletons (n=513)** | | | **Multiples (n=259)** | | |
| Composite outcome | 0.88 | 34/222 (15.3) | 45/242 (18.6) |  | 24/142 (16.9) | 24/117 (20.5) |  |
| Ventilation support (intubation) | 0.87 | 31/216 (14.4) | 48/238 (20.2) |  | 21/139 (15.1) | 18/111 (16.2) |  |
| **Nulliparous vs multiparous women** | ***P* interaction term** | **Nifedipine** | **Atosiban** | **OR (95% CI)** | **Nifedipine** | **Atosiban** | **OR (95% CI)** |
| **Maternal outcomes** |  | **Nulliparous (n=387)** | | | **Multiparous (n=254)** | | |
| GA at delivery (weeks) | 0.81 inner loop failed to converge | 33+1 (30+4-36+5) | 32+1 (30+1-35+0) |  | 36+1 (31+6-38+2) | 34+1 (31+3-38+0) |  |
| Time to delivery (days) | 0.61 inner loop failed to converge | 7 (1-34) | 4 (1-36) |  | 33 (7-53) | 26 (3-50) |  |
| Successful 48 hours of tocolysis | 0.64 | 137/196 (69.9) | 131/191 (68.8) |  | 110/125 (88.0) | 111/129 (86.0) |  |
| **Neonatal outcomes** |  | **Nulliparous (n=469)** | | | **Multiparous (n=302)** | | |
| Composite outcome | 0.83 | 45/224 (20.1) | 50/222 (22.5) |  | 13/140 (9.3) | 19/137 (13.9) |  |
| Ventilation support (intubation) | 0.90 | 35/217 (16.1) | 47/214 (22.0) |  | 17/138 (12.3) | 19/135 (14.1) |  |
| **History vs no history of preterm birth** | ***P* interaction term** | **Nifedipine** | **Atosiban** | **OR (95% CI)** | **Nifedipine** | **Atosiban** | **OR (95% CI)** |
| **Maternal outcomes** |  | **History of preterm birth (n=88)** | | | **No history of preterm birth (n=552)** | | |
| GA at delivery (weeks) | 0.071 inner loop failed to converge | 36+2 (32+6-38+1) | 33+4 (30+2-37+0) | HR 0.54 (0.32-0.90), p=0.018 | 33+4 (31+0-37+1) | 33+0 (30+3-36+1) | HR 0.86 (0.71-1.04), p=0.11 |
| Time to delivery (days) | 0.31 inner loop failed to converge | 30 (8-49) | 14 (3-47) | HR 0.58 (0.34-0.99), p=0.044 | 15 (2-43) | 8 (1-39) | HR 0.87 (0.72-1.05), p=0.15 |
| Successful 48 hours of tocolysis | 0.73 | 39/44 (88.6) | 38/44 (86.4) |  | 208/277 (75.1) | 203/275 (73.8) |  |
| **Neonatal outcomes** |  | **History of preterm birth (n=95)** | | | **No history of preterm birth (n=675)** | | |
| Composite outcome | 0.072 | 2/42 (4.8) | 9/45 (20.0) | 0.18 (0.034-0.92), p=0.039 | 56/322 (17.4) | 60/313 (19.2) | 0.86 (0.15-4.83), p=0.86 |
| Ventilation support (intubation) | 0.85 failed to converge | 4/42 (9.5) | 9/45 (20.0) |  | 48/313 (15.3) | 57/303 (18.8) |  |
| **History vs no history of term birth** | ***P* interaction term** | **Nifedipine** | **Atosiban** | **OR (95% CI)** | **Nifedipine** | **Atosiban** | **OR (95% CI)** |
| **Maternal outcomes** |  | **History of term birth (n=262)** | | | **No history of term birth (n=378)** | | |
| GA at delivery (weeks) | 0.95 | 36+3 (32+5-38+1) | 35+0 (31-6-38+0) |  | 33+0 (30+3-36+2) | 31+6 (30+1-34+4) |  |
| Time to delivery (days) | 0.95 | 34 (9-53) | 31 (4-52) |  | 5 (1-32) | 3 (1-27) |  |
| Successful 48 hours of tocolysis | 0.74 | 120/136 (88.2) | 112/126 (88.9) |  | 127/185 (68.6) | 129/193 (66.8) |  |
| **Neonatal outcomes** |  | **History of term birth (n=330)** | | | **No history of term birth (n=440)** | | |
| Composite outcome | 0.91 failed to converge | 18/164 (11.0) | 15/145 (10.3) |  | 40/200 (20.0) | 54/213 (25.4) |  |
| Ventilation support (intubation) | 0.64 | 22/163 (13.5) | 16/143 (11.2) |  | 30/192 (15.6) | 50/205 (24.4) |  |
| **Neonatal sex*** | ***P* interaction term** | **Nifedipine** | **Atosiban** | **OR (95% CI)** | **Nifedipine** | **Atosiban** | **OR (95% CI)** |
| **Maternal outcomes** |  | **Boys (n=340)** | | | **Girls (n=276)** | | |
| GA at delivery (weeks) | 0.28 | 33+5 (30+5-37+1) | 32+6 (30+1-36+0) |  | 33+6 (31+5-37+5) | 33+2 (30+6-37+1) |  |
| Time to delivery (days) | 0.54 | 16 (2-42) | 6 (1-37) |  | 19 (2-48) | 18 (2-45) |  |
| Successful 48 hours of tocolysis | 0.54 | 129/171 (75.4) | 125/169 (74.0) |  | 109/136 (80.1) | 113/140 (80.7) |  |
| **Neonatal outcomes** |  | **Boys (n=389)** | | | **Girls (n=331)** | | |
| Composite outcome | 0.12 | 27/181 (14.9) | 40/182 (22.0) |  | 24/155 (15.5) | 22/153 (14.4) |  |
| Ventilation support (intubation) | 0.80 | 26/176 (14.8) | 38/176 (21.6) |  | 20/151 (13.2) | 21/149 (14.1) |  |

Data are median (IQR) or n/N (%). OR: odds ratio. MD: median difference. GA: gestational age.

* excludes multiple pregnancies with children from both sexes.

**Appendix S3:** **Sensitivity analysis: two-stage meta-analysis of all studies**

| **Prolongation of pregnancy: 48 hours** |
| --- |


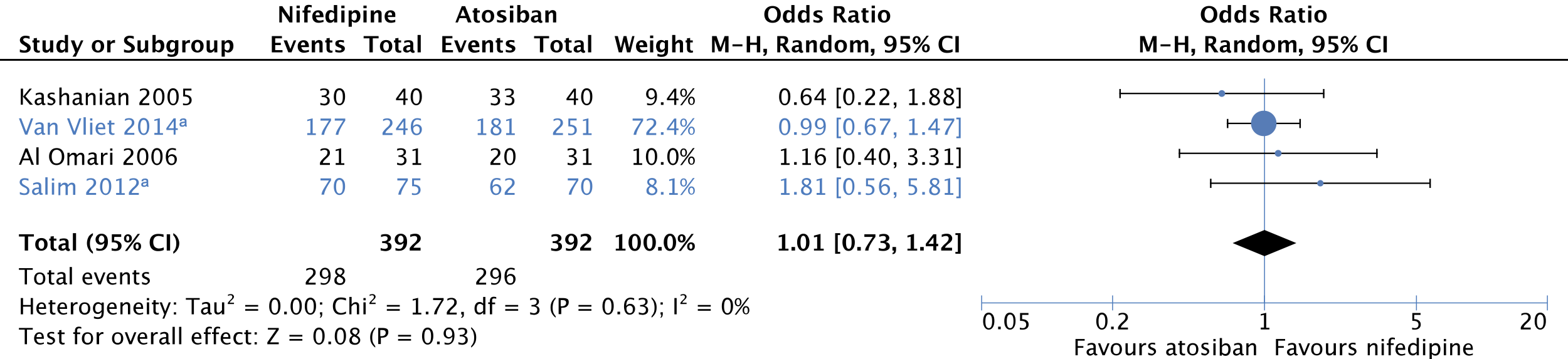


^a^Derived from IPDMA, results may differ from published data

| **Prolongation of pregnancy: 7 days** |
| --- |


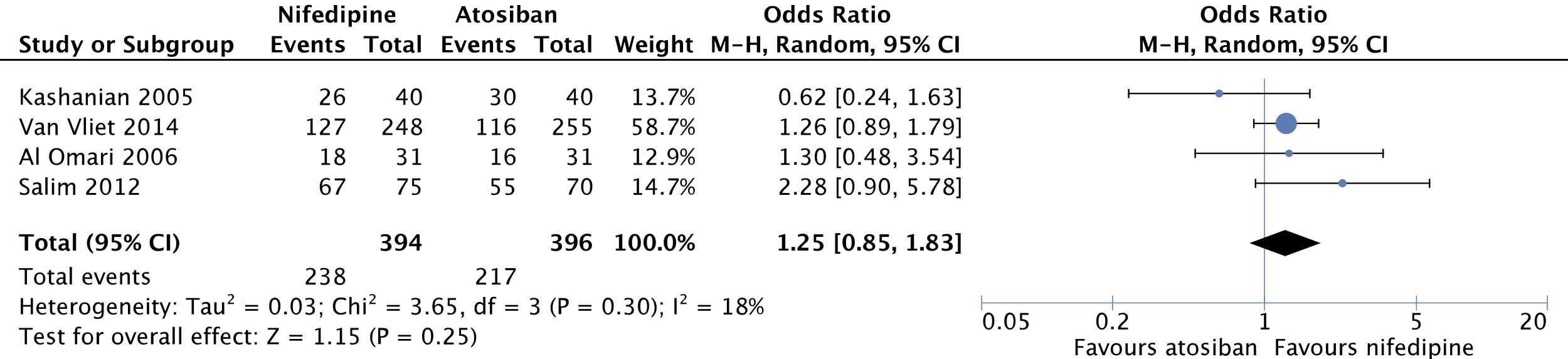


| **Neonatal mortality** |
| --- |


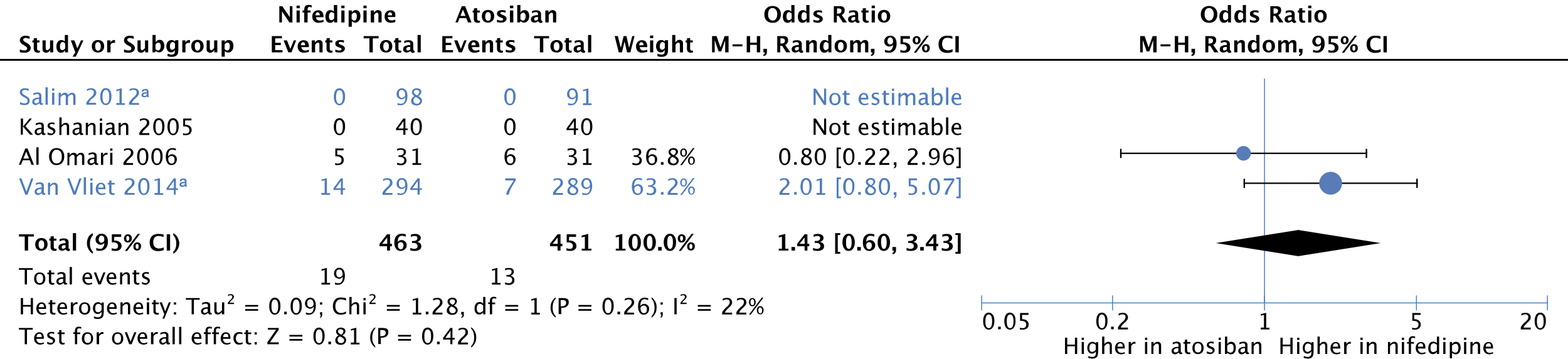


^a^Derived from IPDMA, results may differ from published data
